# Characterisation of intrusive memories and prediction from memory-related genes and cognitive and emotional factors

**DOI:** 10.1038/s41598-025-29775-9

**Published:** 2025-12-22

**Authors:** Laura E. Meine, Linda S. Schaekel, Hanna Thörn, Ulrike Ehlert, Monika Brodmann Maeder, Aristomenes K. Exadaktylos, Roland Bingisser, Andreas Papassotiropoulos, Dominique de Quervain, Isaac Galatzer-Levy, Katharina Schultebraucks, Birgit Kleim

**Affiliations:** 1 Experimental Psychopathology and Psychotherapy, Department of Psychology, University of Zurich, Binzmühlestrasse 14, Zurich, 8050 Switzerland; 2 Department of Adult Psychiatry and Psychotherapy, Psychiatric University Clinic Zurich and University of Zurich, Zurich, Switzerland; 3 Clinical Psychology and Psychotherapy, Department of Psychology, University of Zurich, Zurich, Switzerland; 4 Swiss Institute of Medical Education and Department of Emergency Medicine, Inselspital, University Hospital, University of Bern, Bern, Switzerland; 5 Department of Emergency Medicine, Inselspital, University Hospital, University of Bern, Bern, Switzerland; 6 Emergency Department, University Hospital Basel, University of Basel, Basel, Switzerland; 7 Division of Molecular Neuroscience, Department of Biomedicine, University of Basel, Basel, Switzerland; 8 Research Cluster Molecular and Cognitive Neurosciences, University of Basel, Basel, Switzerland; 9 Psychiatric University Clinics, University of Basel, Basel, Switzerland; 10 Division of Cognitive Neuroscience, Department of Biomedicine, University of Basel, Basel, Switzerland; 11 Department of Psychiatry, NYU Grossman School of Medicine, New York, NY USA; 12 Division of Healthcare Delivery Science, Department of Population Health, NYU Grossman School of Medicine, New York, NY USA

**Keywords:** Emergency medicine, Intrusive memories, Stress, Rumination, Health care, Health occupations, Risk factors, Psychology

## Abstract

Frontline caregivers, such as nurses and physicians, face heightened risk of intrusive memories, a core symptom of posttraumatic stress disorder. Understanding intrusions is key to protecting workers’ mental health and sustaining effective patient care. Emergency department (ED) staff (*N* = 331) were assessed before starting ED work and three and six months later. Baseline measures included demographics, prior trauma, cognitive and emotional processing, and polymorphisms of the memory-related *NR3C1*, *KIBRA*, and *ADRA2B* genes as predictors of intrusions at three months, i.e., peak ED stress exposure. We examined intrusion occurrence, frequency, distress, and content, and investigated whether experiencing intrusions moderated changes in mental health from baseline to follow-ups. Intrusions were prevalent and often concerned potential patient care errors. Being a carrier of the G allele of the BCL1 polymorphism of the *NR3C1* gene and ruminating were associated with intrusions at three months, while cognitive flexibility, emotional suppression, and emotion-focused positive rumination appeared protective. From baseline to three and six months, participants’ anxiety increased, and those experiencing intrusions reported significantly lower work performance and engagement. Our results underline the psychological costs of high-stakes ED settings and suggest improvements to error culture and emotion regulation training as promising avenues to support staff and ultimately patients.

**Supplementary Information:**

The online version contains supplementary material available at 10.1038/s41598-025-29775-9.

## Introduction

Intrusive emotional memories occurring soon after a potentially traumatic event are considered a normal feature of adaptation^1^. They transpire involuntarily, as visual, auditory, tactile or olfactory expressions that are generally characterised as highly emotionally distressing^2^. Intrusive memories can be more or less vivid, ranging from momentary sensory impressions to full flashbacks^3^. Intrusions subside with time in most individuals, but when they persist and continue to be distressing, they are identified as a hallmark feature of posttraumatic stress disorder (PTSD)^4^. Regardless of their persistence, intrusive memories may be associated with high levels of stress and functional impairment^5^.

In emergency departments (EDs), potentially traumatic events occur frequently, including resuscitation attempts, deaths or upset relatives of patients. In a study of 91 emergency nurses who had witnessed “critical incidents”, 65% reported intrusive memories^6^. These included images of suffering or dead patients mixed with loud and chaotic sounds of the ED. Accordingly, 48% of the sample described considerable to extreme stress due to the intrusions. Findings from Zlotnick et al.^5^ reveal that subthreshold PTSD symptoms are associated with similar levels of social and functional impairment as clinically diagnosed PTSD. In line with this, research has shown that intrusive memories interfere with concentration^7^, likely negatively impacting work performance. A concern for healthcare workers is how their symptoms could potentially hamper their ability to provide top-notch patient care^8^. The recent COVID-19 pandemic exacerbated already difficult conditions, as frontline workers such as intensive care unit (ICU) staff faced particularly challenging times. In a study of 709 ICU workers assessed during the peak of the pandemic in 2020, 40% reported PTSD-related symptoms^8^. A recent meta-analysis indicates that healthcare workers in general have a two-fold higher likelihood of developing PTSD compared to the general population^9^. A review examining stress, anxiety, and depression in healthcare professionals during the COVID-19 pandemic in China, Italy, Greece and the United States found high incidence rates^10^. Nurses, female healthcare providers, and those who have less medical experience or suffer from pre-existing mental health issues were found to be more likely to be affected by significant mental distress^11^. Moreover, healthcare workers may show emotional pain related to adverse events they were not directly involved in (second victim experiences) with a recent meta-analysis reporting 20% were experiencing intrusions^12^.

These findings underline the need for a more thorough assessment of PTSD symptoms, specifically intrusive memories, in this population. Although PTSD is very heterogeneous^13^, intrusions form a core symptom, formalised as Criterion B in the diagnosis of PTSD^4^ and constitute a proximal, mechanistically meaningful factor that may directly impact work performance and well-being in healthcare personnel and first responders, beyond the diagnostic threshold of PTSD. A focus on intrusive memories may help better understand risk factors and mechanisms, a prerequisite for accurate risk identification and the development of effective, personalised, and timely interventions to prevent persistent psychopathology. The promise of data-driven prediction models for early disease detection and individualised treatment options has been highlighted in many recent studies^14,15^. In the US, researchers could leverage data routinely collected from patients admitted to the ED after a traumatic event to develop algorithms that can forecast the risk of developing PTSD^16,17^. However, such analyses typically require large sample sizes which are not always readily obtainable. Alternatively, it can be helpful to constrain the predictor set by selecting variables based on prior literature and theoretical considerations. With regard to intrusive emotional memories, there are reviews highlighting certain neural, hormonal, psychophysiological, and cognitive factors which may influence the development of symptoms^18,19^. A focus on symptoms rather than diagnoses has translational benefits and can lead to scalable treatment^18^. Marks et al.^19^ synthesised evidence on pre-existing vulnerabilities such as elevated stress levels, chronic consumption of drugs or alcohol, lower working memory capacity, female gender, and specific genetic variants. In addition, they discussed factors directly related to the traumatic event such as high negative emotional arousal, increased activity of the noradrenaline system, as well as sensory-perceptual processing such as dissociation or decontextualisation which render the experience of intrusive memories more likely. Given the importance of memory consolidation and retrieval, post-event factors such as negative appraisal style, the tendency to ruminate, and suppression of negative thoughts also play a crucial role.

Based on these findings, we investigated characteristics and predictive factors of intrusive emotional memories and associations with mental health and work performance in a longitudinal study of ED personnel. The aim was three-fold: First, we sought to provide a more thorough understanding of how intrusive memories present in this population by describing not only rates of participants who experienced intrusions, but also characterising their frequency, quality, and content. Second, we investigated a set of theory-derived baseline factors, comprising memory-related genes, cognitive and emotional processing (see Supplementary Table 1 for an overview) to identify the most relevant factors for the development of intrusive memories and the level of intrusion-related distress. Third, we probed links between intrusion occurrence, mental health, and work problems by testing whether experiencing intrusions was positively associated with symptoms of depression and anxiety as well as work performance and engagement.

## Results

### Sample descriptives

At baseline (T0), participants rated themselves as “somewhat experienced” in emergency medicine (*M* = 2.09, *SD* = 0.79; scale ranging from 1 = very inexperienced to 4 = very broad experience). Out of ten possible prior traumatic events, they had experienced on average between one and two (*M* = 1.53, *SD* = 1.44).

### Intrusive emotional memories

Of the *N* = 331 participants, 193 responded to the follow-up survey and provided data on intrusions and other indices at three months after starting work in the ED (T1), and 197 at six months (T2), providing data on intrusions and other indices. These subsamples formed the basis for the analyses. A substantial proportion of those participants reported experiencing intrusive memories at T1 and T2, see Table 1. At T1, about 40% noted intrusions, the majority of which were characterised as thoughts, followed by a mixture of thoughts, feelings, and sensory experiences, with feelings alone making up the smallest percentage. This pattern was similar at T2, with 35% of participants reporting intrusions. Nearly a third of those participants who had initially reported intrusive memories were still experiencing them at six months (*n* = 28). At both time points, close to three quarters of participants affirmed their intrusions had flashback quality, i.e., they were re-experiencing events as if they were occurring in the present. Regarding the frequency of intrusions in the past week, participants reported on average almost 2 intrusions at T1, and about 1.5 at T2. Ratings of intrusion-related distress indicate that participants felt moderately burdened at both time points. We also observed low rates of impairment.

**Table 1:** Characteristics of intrusive memories reported at 3- and 6-months follow-up.

Variable	T1 (3 months) (*N* = 193 provided data)	T2 (6 months) (*N* = 197 provided data)
*n* participants reported intrusions (%)	82 (42.49)	69 (35.03)
*n* intrusion type (%)		
Thoughts	41 (50.00)	30 (44.78)
Feelings	11 (13.41)	13 (19.40)
Sensory experience	0 (0.00)	1 (1.49)
Mixture	30 (36.59)	23 (34.33)
*n* intrusions with flashback quality (%)	61 (74.39)	50 (73.53)
Frequency in the past week, *M* (*SD*), range	1.76 (1.49), 0–7	1.39 (1.40), 0–7
Level of distress, *M* (*SD*), range	1.96 (0.71), 1–4	2.09 (0.74), 1–4
Intrusion-related impairment, *M* (*SD*), range	1.51 (0.51), 1–3	1.36 (0.46), 1–3

Descriptive statistics were calculated after exclusion of missing values, percentages therefore denote valid percentages.

### Intrusion content

**Fig. 1:**
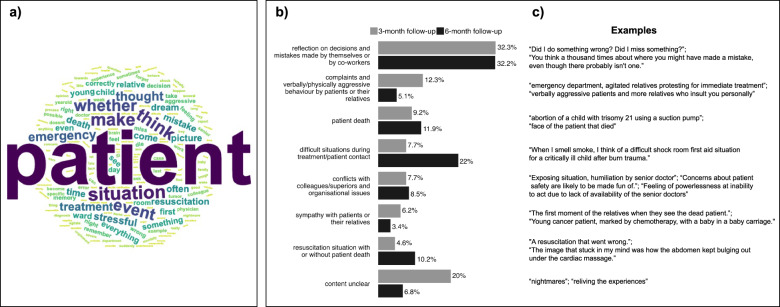
Reports of intrusion content. (**a**) Word cloud based on reports of intrusion content at 3- (T1) and 6-months follow-up (T2), showing words in decreasing size by frequency; (**b**) percentages of reported intrusion content at T1 and T2 by category with (**c**) sample reports from the dataset.

About a third of intrusions at T1 and T2 were related to decisions and mistakes made by participants or their co-workers. These constituted mainly thoughts about (possible) mistakes in the treatment process and forgetting important tasks. A few such reports included sensory experiences (e.g., “The patient files always come back to me vividly, with the question: Did I do everything right?”) or feelings of worry or guilt. At T1, the second most common intrusions regarded complaints and verbally/physically aggressive behaviour by patients or their relatives, followed by the death of a patient. In contrast, at T2, the second most common intrusion category concerned difficult situations during treatment/patient contact. Examples for this included treatment of severe burns and terminally ill patients. Similarly, intrusions about resuscitation situations with or without patient death were reported about twice as often at T2 compared to T1. At both time points, about 8% of intrusions were related to conflicts with colleagues/superiors and organisational issues. The participants reported, among other things, troubling interactions and feeling helpless in the face of organisational failure or staff shortages. Intrusions concerning sympathy with patients or their relatives were noted twice as often at T1 (6.2%) compared to T2 (3.4%). For some of the intrusions, the content remained unclear because participants merely reported “nightmares”, “reliving the experiences”, or “images that one often cannot forget quickly”. Figure 1a shows words most frequently used in intrusion reports, with “patient” as the most frequently occurring word, and Fig. 1b displays the percentages of categories of reported intrusion content at both time points (65 reports at T1 and 60 reports at T2), including example reports.

### Relevance of memory-related genes, cognitive and emotional processing for intrusion occurrence, frequency, and related distress

We next investigated the predictive value of our baseline measures for the occurrence of intrusions, their frequency in the past week, and the level of related distress at T1. While intrusion-related distress ratings were fully available from the 82 participants who had experienced intrusions at T1, 36 of these did not indicate intrusion frequency and the majority of those who did provide this data reported only a single intrusion in the past week (*Median* = 1). Consequently, this outcome did not offer much information beyond the binary assessment of whether intrusions occurred or not. We therefore decided against setting up a regression model for intrusion frequency. After excluding participants missing information on intrusion occurrence (our main outcome of interest), data on gene polymorphisms, or sex (the latter two constituting predictors where missings cannot be readily imputed), our remaining sample numbered 110 participants with 2.42% of values still missing in the predictor set. No patterns in missingness emerged and Little’s test was not significant (*χ*^*2*^(162) = 176, *p* = .211). Therefore, we assumed the missing values to be MCAR and imputed them. There was also no evidence of multicollinearity among predictors (all correlation coefficients *r* < .70; Supplementary Table 2).

#### Features associated with intrusion occurrence

**Fig. 2:**
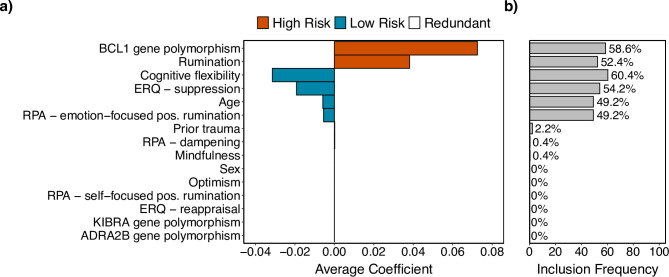
Feature importance and inclusion frequencies of predictors of intrusion occurrence. (**a**) Average coefficients and (**b**) inclusion frequencies across 500 repeats least absolute shrinkage and selection operator (LASSO) regression.

The LASSO regression revealed being a carrier of the G allele of the BCL1 polymorphism and the tendency to ruminate as risk factors most strongly associated with the experience of intrusions at T1. Cognitive flexibility, suppression of negative emotions, emotion-focused positive rumination (e.g., savouring the moment or noticing feeling full of energy) as well as older age emerged as protective factors. Other predictors were not selected (see Fig. 2a). Examination of inclusion frequencies (see Fig. 2b) showed that cognitive flexibility, the BCL1 polymorphism, emotional suppression, and rumination were selected most consistently across LASSO repeats with 50–60%, followed by emotion-focused positive rumination and age (both about 50%). 

### Features associated with intrusion-related distress

Given the relatively small subsample of *n* = 82 participants who reported experiencing intrusions at T1 and rated intrusion-related distress, with 33 not providing saliva samples for analyses of gene polymorphisms, we only tested cognitive flexibility, rumination, and emotional suppression as predictors of intrusion-related distress at T1, i.e., the self-report features most consistently selected as important for the development of intrusive memories in the LASSO regression. These predictors appeared conceptually relevant to subjective distress, and we thus did not need to exclude any further cases. With only three predictors, model complexity was sufficiently low to allow for standard multiple linear regression without regularisation. The analysis revealed no significant effects for any of the predictors (see Supplementary Table 3).

### Testing associations between intrusion occurrence and depressive symptoms, anxiety, and work performance and engagement

**Fig. 3:**
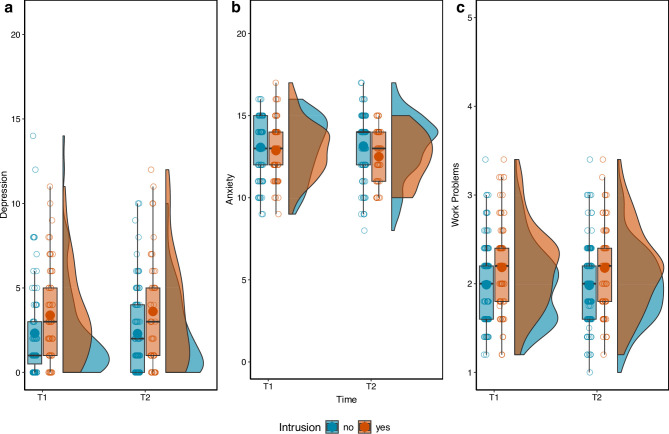
Mental health and work problems by intrusion occurrence at T1 and T2. (**a**) Depressive symptoms, (**b**) anxiety symptoms, and (**c**) work performance and engagement. Filled points overlaid on boxplots show mean values.

While levels of depression and work-related adjustment were relatively stable over the study period, participants showed a significant increase in anxiety from T0 to follow-ups (see Supplementary Table 4). Focusing on inter-individual differences, we tested whether participants who experienced intrusions reported more symptoms of depression and anxiety and more work problems while working in the ED. There were no significant associations between intrusion occurrence and mental health (depressive symptoms and anxiety) at either T1 or T2 when controlling for baseline mental health symptoms. We mainly observed positive associations between baseline mental health and symptom severity at follow-up. However, participants who experienced intrusions at T2 reported significantly more problems concerning work performance and engagement at T2 (*β* = 0.25, *t* = 3.21, *p* = .002). This effect remained significant even when controlling for baseline depressive symptoms and anxiety. Sensitivity analyses (Supplement 3) indicated that, given the available number of observations, only moderate-to-large effects could be reliably detected. The observed effects of intrusion occurrence on depression and anxiety were small and below this detectable range, suggesting that these associations may have been too subtle to detect reliably in the present sample. See Supplementary Tables 5–10 for detailed results of all models, including effect sizes, and Fig. 3 for a visualisation.

## Discussion

We characterised intrusive emotional memories in ED personnel—a group frequently exposed to stress and potentially traumatic events. Intrusions were prevalent in this sample, which is in line with previous reports^6,8^, while intrusion-related distress and impairment was reported as moderate. The majority of intrusions concerned real or imagined mistakes during treatment. Compared to three months after starting work in the ED, at six months, participants reported fewer intrusions related to emotional interactions with patients or patients’ relatives. At this later follow-up, intrusions more often concerned difficult treatment situations. These findings may reflect participants growing more used to challenging conversations with patients or relatives as emergency care becomes more routine over time. Participants may also have developed effective strategies for dealing with conflicts. In turn, they may become more occupied with the treatment, having gained more experience and skills and, correspondingly, greater responsibility for a patient’s life. Future studies could examine individual trajectories of intrusion content to better delineate accumulation of different traumatic events and the persistence of intrusions for single events in healthcare staff.

The finding that participants predominantly experienced intrusions related to perceived mistakes points to the importance of positive error culture in medical practice and especially in the ED. Errors occur daily and in every aspect of life. Error management involves effectively addressing errors with the aim of minimising future occurrences, mitigating negative consequences, and promptly handling the aftermath of errors^20^. Error management training is a potent intervention to enhance performance on complex transfer tasks according to a recent meta-analysis^21^. The importance of such interventions is underlined by recent results showing that only a third of medical staff interviewed believed that errors were appropriately handled in their hospitals^22^. A significant portion of ICU staff did not acknowledge their own errors, and many reported finding it challenging to discuss mistakes. In a study conducted among faculty and resident physicians in the United States^23^, most participants expressed a willingness to report hypothetical errors causing harm, while only a minority actually reported errors. These findings highlight the need to address barriers to critical incident reporting and improve awareness of reporting procedures among physicians to enhance patient safety and reduce the emotional impact on the physicians. Asakawa et al.^24^ described various forms of self-disclosure which form part of medical residents’ recovery and learning processes following medical errors. Understanding these processes is crucial for fostering a “culture of sharing errors” in hospitals as high reliability organisations^24^.

Being a carrier of the G allele of the BCL1 polymorphism of the *NR3C1* gene emerged as a risk factor for the occurrence of intrusive memories, which is in line with previous results^25^ and with evidence linking this gene to PTSD^26^. Genetic and epigenetic markers within *NR3C1* are also associated with response to trauma-focused cognitive behavioural therapy^27^. Genotyping could help detect vulnerable individuals who may then receive targeted interventions to better process potentially traumatic events, e.g., as envisioned in^28^, ideally preventing the development of PTSD symptoms, such as intrusive memories.

The tendency to ruminate also emerged as a risk factor for intrusions. Participants most often reported experiencing intrusive memories in the form of thoughts or a mixture of thoughts, feelings, and sensory experiences. This points to the question of how to distinguish between ruminative thoughts and intrusive thoughts. Ruminative thoughts and intrusive thoughts are both types of repetitive, unwanted cognitions that can cause distress, but they differ in their underlying characteristics and focus. Ruminative thoughts typically involve persistent and repetitive thinking about past events, focusing on negative aspects and their potential causes and consequences^29^. On the other hand, intrusive thoughts are characterised by sudden, involuntary, and intrusive mental images, ideas, or impulses that can be distressing^1^. In the present study, the majority of participants described their intrusions as having flashback character, indicating the presence of intrusions. However, it remains plausible that those with intrusions also engaged in rumination. Both share underlying cognitive mechanisms, such as heightened reactivity and impaired executive control^30^ and understanding their interplay is critical for developing targeted interventions.

Our findings underscore the importance of cognitive flexibility and emotion regulation as useful strategies for navigating the fast-paced ED environment. Although emotional suppression has been regarded as maladaptive for mental health outcomes^31^, it was negatively associated with the occurrence of intrusive memories. It may be that participants who generally had a greater ability to regulate negative emotions through suppression were protected from developing intrusions. It would be particularly insightful to examine ecological momentary assessments comparing suppression both within and outside of work, as well as during and outside of stressful periods. Research on memory suppression^32^ suggests that suppressing unwanted memories can reduce their unintended influence, which may be particularly relevant in demanding environments where intrusions can be disruptive. In high-stakes situations such as resuscitation attempts, where maintaining a clear head is critical, suppression may serve as an adaptive strategy by preventing distressing memories from interfering with performance.

The identified risk and protective factors were not predictive of intrusion-related distress. It may be that other factors influence whether the experience of intrusive memories goes along with heightened levels of distress. However, the small sample, the relatively low distress ratings and rather infrequent intrusions may also explain the lack of significant effects. Although most participants reported infrequent intrusive memories, these experiences were functionally relevant: participants who reported intrusions showed significantly lower work performance and engagement. Future research could examine point-prevalence and identify factors contributing to higher intrusion frequency, which may help clarify vulnerability profiles and inform targeted interventions for individuals at greater risk. Intrusion occurrence was not significantly associated with depression and anxiety symptoms. However, sensitivity analyses indicated that our study was powered to detect only moderate-to-large effects, and smaller associations may have gone undetected. Participants generally showed low depressive symptoms but significantly increased in anxiety with the average score at three and six months above the established cut-off of 8^33,34^. Entering a high-demand clinical environment likely heightens vigilance and worry (consistent with elevated anxiety). Given that the sample consisted of regular ED personnel, participants likely represented a stressed but generally healthy working population. Accordingly, effects of intrusions on mental health outcomes may have been subtle. It also remains possible that effects of intrusions on mental health manifest later than six months, especially if individuals experience multiple potentially traumatic events and if work problems persist. Given the enormous responsibility ED personnel carry, it is crucial to prevent and mitigate effects on work performance and engagement to support health care professionals themselves as well as their patients.

Our study supports reports that ED personnel operate under considerable strain and are therefore at increased risk of developing mental disorders^6,8^. The Swiss press recently published several articles highlighting the problematic working conditions faced by physicians across all specialties, reporting that labour laws were routinely flouted, residents frequently work overtime, suffer from burnout symptoms, and express frustration over a lack of support from superiors and hospital management^35,36^. While many departments already struggle with a shortage of physicians^37,38^, a third of medical students admit reconsidering their choice of profession following first practical experiences^39,40^. Medical students, being among the least experienced healthcare workers, are at heightened risk for psychological distress^41^.

There has been recent progress in increasing the healthcare workforce globally^42^, but more sustainable approaches to build and maintain a good system are urgently needed. Efforts to ameliorate the difficult working conditions for healthcare staff, particularly in the ED, should be broad and involve policymakers, hospital managers, doctors, teaching staff, etc. Improvements in team and error culture as well as the provision of support and resources to individual workers are two key avenues. Regarding the latter, a recent meta-analysis evaluating lab-analogue-trauma studies confirmed that there are efficacious techniques which significantly reduce the frequency of intrusive memories and ameliorate related distress^43^. One promising example involves mental imagery thought to interfere with the trauma memory, rendering it less intrusive. Ramineni et al.^44^ recently tested such a digital imagery-competing task intervention for ICU staff facing trauma during the COVID-19 pandemic. The immediate intervention group showed significantly fewer intrusive memories related to trauma compared to the delayed intervention group. Interviews with healthcare workers also confirmed their acceptance of such visuo-spatial tasks^45^, which offer an easy, brief, and effective intervention.

Our study has some limitations. First, the sample size was rather small for predictive modelling and missing values required us to exclude a number of cases from analyses. However, given that the data was collected via anonymous survey as part of routine assessments in medical care, our dataset represents the typical case. While large samples are crucial for robust predictions, models should be tested on real-life datasets to not only be useful under perfect conditions in the lab. We acknowledge that our findings need to be replicated in independent research. Second, we lacked a formal diagnostic assessment of PTSD symptoms which would have allowed for better characterisation of severity and to rule out the possibility that some participants already had symptoms that may have predisposed them to experiencing intrusions related to stressful experiences in the ED. Third, some of the questionnaires showed only acceptable internal consistency. Fourth, we did not include a long-term follow-up but would recommend doing so in future studies. Fifth, rather than conducting a genome-wide-association-study, we derived specific gene polymorphisms through theoretical considerations and based on expert opinion. Finally, due to time constraints, we were unable to comprehensively assess reported intrusion content and did not have sufficient data for more in-depth text analyses. Ecological momentary assessment has proven useful for obtaining details on day-to-day dynamics of individuals’ experience of intrusive thoughts^46,47^.

## Conclusion

Our study highlights the prevalence and characteristics of intrusive emotional memories among healthcare staff and their negative effects on work performance. The findings offer starting points for system-level improvements, e.g., to hospital error culture, as well as for targeted prevention and intervention strategies aimed at supporting medical personnel by enhancing cognitive flexibility and emotion regulation training. Addressing these challenges proactively may enhance well-being and resilience of healthcare professionals but also improve the quality of care to patients, fostering a healthy and sustainable healthcare environment.

## Materials and methods

### Participants and procedure

ED staff (*N* = 331) working at a Swiss University Hospital took part in this study (see Table 2 for detailed demographics). Most participants were medical staff or physicians in training with on average 4.4 years of medical work experience. They were initially assessed within two weeks of starting work in the ED (T0) and followed up three (T1) and six months later (T2). At baseline, participants provided self-report data on demographics, medical experience, prior trauma, cognitive and emotional processing, and mental health. We also collected saliva samples for assessment of selected gene polymorphisms. At both follow-ups, participants completed questionnaires on intrusive emotional memories, mental health, and work problems.

Participation was voluntary and all participants provided written informed consent prior to testing. Ethics approval was obtained from the Cantonal Ethics Committee of Zurich (No. 2010 − 0517) and all procedures contributing to this work comply with the Helsinki Declaration of 1975, as revised in 2008.

**Table 2:** Demographics (assessed at baseline).

Variable	
*N* females (%)	153 (64.29)
Age in years, *M* (*SD*), range	29.14 (4.84), 22–53
Home country, *N* (%)	
Switzerland	146 (61.60)
Other European country	87 (36.71)
Country outside Europe	4 (1.69)
Professional role, *N* (%)	
Medical staff	138 (58.23)
Nursing staff	19 (8.02)
Physician in training	80 (33.76)
Medical education in years, *M* (*SD*)	7.06 (3.24)
Medical work experience in years, *M* (*SD*)	4.40 (3.16)
Total number of distinct potentially traumatic events experienced, *M* (SD)	1.53 (1.44)

Descriptive statistics were calculated after exclusion of missing values, percentages therefore denote valid percentages.

### Measures

#### Prior trauma

We derived ten potentially traumatic events from the Posttraumatic Diagnostic Scale (PDS)^48^ and calculated the total number of distinct trauma types participants had previously experienced. The checklist comprised accidents, natural disasters, assault, sexual abuse, war, imprisonment, and life-threatening illness.

#### Cognitive and emotional processing

Cognitive processing was assessed via the Cognitive Flexibility Questionnaire (CFQ)^49^ and rumination was measured with select items from the Response Styles Questionnaire (RSQ)^50^. To probe rumination also in the context of positive mood, participants filled in the Responses to Positive Affect (RPA) Questionnaire^51^. We assessed participants’ ability to regulate emotions using the Emotion Regulation Questionnaire (ERQ)^52^ and measured dispositional mindfulness with the Mindfulness Attention and Awareness Scale (MAAS)^53,54^. Finally, we indexed optimism using the revised Life Orientation Test (LOT-R)^55,56^. See Supplement 1 for more details on these questionnaires, including item examples and internal consistency scores.

#### Intrusive emotional memories

We used an adapted version of an established intrusion questionnaire^57^. Participants reported whether or not they had experienced any intrusions, indicated their frequency in the past week and described their specific content and type (i.e., thoughts/feelings/sensory experiences/mixture). They used a 4-point scale to rate the extent to which the memory was replayed in their mind’s eye (1 = not at all, 2 = a little, 3 = very, 4 = absolutely) and we binarised these data to indicate whether an intrusion had flashback quality or not (ratings of 1 = no flashback quality; ratings of 2–4 = flashback quality). Intrusion-related distress was rated on a scale ranging from 1 = not burdensome to 4 = extremely burdensome. We also inquired about possible nightmares, depressive symptoms, anxiety, desire to withdraw from others, mood swings, and guilty conscience, using a scale from 1 (not at all) to 4 (often). These additional items were averaged to index overall intrusion-related impairment.

#### Mental health and work-related adjustment

Participants completed the Hospital Anxiety and Depression Scale (HADS)^34,58^ at all time points. This scale consists of 14 items, half of which assess symptoms of depression and the other half symptoms of anxiety. Items are rated as 0 to 3 denoting either the frequency with which symptoms are experienced or the intensity. In this study, both scales showed acceptable internal consistency based on participants’ assessments at baseline (Cronbach’s α = 0.74 and α = 0.76 for depression and anxiety, respectively). To probe functioning at work, we selected five items from the Social Adjustment Scale^59,60^ which focuses on the past two weeks. Participants rated how well they accomplished their work, whether they were unhappy with their performance, whether they had conflicts with colleagues or superiors, whether they were annoyed with their work and whether they found it interesting. Items were rated on a 5-point scale with response options depending on the item (e.g., “no conflict” to “a lot of conflict” or “never annoyed” to “always annoyed”). We calculated a sum score where higher values indicate more problems at work. The scale showed acceptable internal consistency based on participants’ ratings at baseline (Cronbach’s α = 0.61).

#### Genotyping

For details on the collection and processing of saliva samples, see Supplement 2. We focused on gene polymorphisms that have been consistently associated with differences in memory processes involved in the development of intrusive memories. Carriers of the G allele of BCL1, a single-nucleotide polymorphism of the glucocorticoid receptor (GR) gene (*NR3C1*), reportedly show heightened GR sensitivity^61^ and are more prone to developing intrusive trauma memories^25^. A deletion variant of the gene encoding the α2-adrenergic receptor (*ADRA2B*) has been associated with greater engagement of the amygdala in response to stress^62^ and emotional content, with correspondingly amplified formation of emotional memories^63,64^ but a decreased ability to access the appropriate memory system in stressful situations^65^. The *WWC1* gene, encoding the kidney and brain expressed protein (*KIBRA*) plays an important role in episodic and working memory performance^66,67^ and has been linked to PTSD development^68^.

We tested whether observed genotype frequencies aligned with frequencies that would be expected if randomly sampling from a population. The exact test for departures from Hardy-Weinberg equilibrium (HWE) showed that the genotype distribution for BCL1 and *KIBRA* was in HWE (*χ*^*2*^ = 0.26, *p* = .643 and *χ*^*2*^ = 1.67, *p* = .167, respectively). However, for *ADRA2B*, the data deviated from HWE (*χ*^*2*^ = 8.30, *p* = .006), indicating overrepresentation of non-deletion homozygotes (LL = 90) and deletion homozygotes (SS = 34) and underrepresentation of heterozygotes (LS = 71) in our sample.

Due to the small number of homozygous carriers of the G allele of BCL1, the deletion variant *ADRA2B*, and the T allele of *KIBRA*, we grouped these together with heterozygotes. In line with previous studies^64,69^, we thus compared carriers with non-carriers.

### Data analysis

Data were processed and analysed in R (version 4.4.1; https://r-project.org*).*

We used descriptive statistics to characterise self-reported intrusive emotional memories by describing occurrence rates, type, frequency, and level of related distress at T1 and T2.

To analyse intrusion content, two independent raters grouped participants’ reports into categories based on guidelines on thematic analysis specified by Braun & Clarke^70^. An initial set of themes was developed based on prior literature^6,71^ and adapted after reading and re-reading participants’ reports. This process yielded the following eight categories that best capture themes observed in the data: 1) reflection on decisions and errors made by themselves or by co-workers, 2) difficult situations during treatment/patient contact, 3) conflicts with colleagues/superiors and organisational issues, 4) complaints and verbally/physically aggressive behaviour by patients or their relatives, 5) resuscitation situation with or without patient death, 6) patient death, 7) sympathy with patients or their relatives, 8) content unclear. Given the relatively short reports (1–43 words), we focused on the type of situations remembered and examined frequencies of themes rather than conducting a more thorough text analysis. We assessed inter-rater reliability in categorising intrusion reports using Cohen’s kappa^72^, revealing on average 80% agreement (T1 reports: *κ* = 0.75, 95% CI [0.64, 0.87]; T2 reports: *κ* = 0.76, 95% CI [0.64, 0.88]). To provide examples, reports of intrusion content were translated from German to English using DeepL machine translation (http://www.deepl.com/en/translator) via the “deeplr” package^73^. For visualisation in the form of a word cloud, we removed punctuation, numbers, and English stop words and lemmatised words using the “tm"^74^ and “textstem”^75^ packages respectively.

To investigate the relevance of memory-related genes, cognitive and emotional processing for the development of intrusive memories, we evaluated the predictive value of the following 15 predictors selected based on previous research and theoretical considerations: BCL1, *ADRA2B*, and *KIBRA* polymorphisms, prior trauma, cognitive flexibility, rumination, dampening, self-focused, and emotion-focused positive rumination, cognitive reappraisal, expressive suppression, mindfulness, optimism as well as age and sex. For this analysis, we exclusively focused on T1 at which time participants were three months into their work experience in the ED. By then, they were fully integrated into ED teams, carrying significant responsibility in highly stressful situations, but had not yet developed the level of routine that may be expected by six months. The T1 assessment therefore best captured the window of heightened acute stress, when intrusions may occur. We excluded cases missing our outcome of interest (occurrence of intrusions) and participants who did not provide saliva samples for genomic analyses or information about their sex as these data cannot be readily imputed. We then inspected remaining missing values in the predictor set and conducted Little’s test to check whether these were missing completely at random (MCAR)^76^. Missing values were subsequently estimated through multiple imputation by chained equations (MICE)^77^ using the “mice” package^78^ with the default of five imputations as we only had a moderate amount of missing data. All variables (including outcomes) were included as predictors in the imputation process. Since missings were restricted to continuous variables, all were imputed through predictive mean matching. We assessed convergence across 30 iterations, visually inspected distributions of observed and imputed variables, and verified that imputed values fell within the range of plausible values for each predictor. To check for multicollinearity, we evaluated correlations between predictors. Due to missing data in our outcome, the number of participants who reported experiencing intrusive memories was lower than expected, precluding a simple logistic regression with many predictors. To deal with this “large *p*, small *n*” issue, we employed least absolute shrinkage and selection operator (LASSO) regression which facilitates feature selection by adding a penalty term λ that encourages sparsity where some coefficients are forced to be exactly zero^79^. The optimal λ was selected via 10-fold cross-validation as that of minimum mean cross-validated error. Categorical predictors were binarised and continuous variables z-standardised. We applied LASSO regression to identify variables most strongly associated with intrusion occurrence out of a set of theory-derived, partly interrelated constructs. Specifically, we employed grouped adaptive LASSO^80^ on all imputed datasets with 500 repeats using the “miselect” package^81^. We then examined average coefficients to gauge the direction of association while considering inclusion frequencies across all repeats as a measure of variable selection stability^82,83^. We conducted an exploratory linear regression with the three self-report variables most consistently selected in the LASSO regression to predict intrusion-related distress in the subset of participants who had experienced intrusions.

In a final analysis, we tested whether the experience of intrusions was positively associated with mental health and work problems. We set up two separate multiple regression models with depression or anxiety scores at T1 as the outcome and intrusion occurrence at T1 (yes/no) as the predictor. Baseline depression or anxiety scores, age, and sex were included as covariates. Models testing associations between intrusion occurrence and mental health problems at T2 were constructed accordingly. Additionally, we examined links between the experience of intrusions and work performance and engagement. Since the latter were only reported at T1 and T2, we only tested associations between intrusion occurrence at T2 and work problems at T2, controlling for work problems at T1, age, and sex. To account for skewed data and outliers, we performed robust linear regression using the “robustbase” package^84^.

## Supplementary Information

Below is the link to the electronic supplementary material.

Supplementary Material 1

## Data Availability

Data and code are available on the Open Science Framework: https://osf.io/wnpbr/.
