# Supplementary material for: Characterisation of intrusive memories and prediction from memory-related genes and cognitive and emotional factors
Source: Sci Rep. 2025 Dec 22;15:45025. doi: 10.1038/s41598-025-29775-9 (PMC12749977; doi:10.1038/s41598-025-29775-9)
Supplement: Supplementary file 1 — Supplementary Material 1 [file 41598_2025_29775_MOESM1_ESM.pdf]

Supplement

Characterisation of Intrusive Memories and Prediction from Memory-Related Genes and Cognitive and Emotional Factors

Laura E. Meine[1,2], Linda S. Schaekel[1,2], Hanna Thörn[1,2], Ulrike Ehlert[3], Monika Brodmann Maeder[4], Aristomenes K. Exadaktylos[5], Roland Bingisser[6], Andreas Papassotiropoulos[7,8,9], Dominique de Quervain[8,9,10], Isaac Galatzer-Levy[11], Katharina Schultebraucks[11,12], Birgit Kleim[1,2]

[1]Experimental Psychopathology and Psychotherapy, Department of Psychology, University of Zurich, Zurich, Switzerland
[2]Department of Adult Psychiatry and Psychotherapy, Psychiatric University Clinic Zurich and University of Zurich, Zurich, Switzerland
[3]Clinical Psychology and Psychotherapy, Department of Psychology, University of Zurich, Zurich, Switzerland
[4]Swiss Institute of Medical Education and Department of Emergency Medicine, Inselspital, University Hospital, University of Bern, Bern, Switzerland
[5]Department of Emergency Medicine, Inselspital, University Hospital, University of Bern, Bern, Switzerland
[6]Emergency Department, University Hospital Basel, University of Basel, Basel, Switzerland
[7]Division of Molecular Neuroscience, Department of Biomedicine, University of Basel, Basel, Switzerland
[8]Research Cluster Molecular and Cognitive Neurosciences, University of Basel, Basel, Switzerland
[9]Psychiatric University Clinics, University of Basel, Basel, Switzerland
[10]Division of Cognitive Neuroscience, Department of Biomedicine, University of Basel, Basel, Switzerland
[11]Department of Psychiatry, NYU Grossman School of Medicine, New York, NY, USA
[12]Division of Healthcare Delivery Science, Department of Population Health, NYU Grossman School of Medicine, New York, NY, USA

Supplementary Information ... 3
Supplement 1. Details on assessment of cognitive and emotional processing ... 3
Supplement 2. Details on saliva sampling and genotyping ... 4
Supplement 3. Sensitivity analyses ... 4
Supplementary Tables ... 5
Supplementary Table 1. Overview of included predictors ... 5
Supplementary Table 2. Zero-order correlations between predictors of intrusion occurrence ... 6
Supplementary Table 3. Predicting intrusion-related distress at 3-months follow-up ... 7
Supplementary Table 4. Mental health and work problems by time point ... 7
Supplementary Table 5. Association between depressive symptoms at T1 and intrusion occurrence at T1 ... 8
Supplementary Table 6. Association between depressive symptoms at T2 and intrusion occurrence at T2 ... 8
Supplementary Table 7. Association between anxiety symptoms at T1 and intrusion occurrence at T1 ... 9
Supplementary Table 8. Association between anxiety symptoms at T2 and intrusion occurrence at T2 ... 9
Supplementary Table 9. Association between work problems at T2 and intrusion occurrence at T2 ... 10
Supplementary Table 10. Association between work problems at T2 and intrusion occurrence at T2 with baseline mental health as covariates ... 10

Supplementary Information

Supplement 1. Details on assessment of cognitive and emotional processing

Cognitive processing was assessed via the Cognitive Flexibility Questionnaire (CFQ; Martin & Rubin, 1995). This scale consists of 12 items which measure awareness of different options in any given situation, willingness to adapt, and self-efficacy in demonstrating flexibility. Participants responded to each item on a 6-point Likert scale, resulting in a sum score ranging from 12-72, with higher scores indicating greater cognitive flexibility. Internal consistency was close to acceptable in the present study, Cronbach's $\alpha = 0.68$. Additionally, six items from the Response Styles Questionnaire (RSQ; Nolen-Hoeksema, 1991) were selected to measure rumination. These included thinking about one's shortcomings, failings and mistakes, thinking about feeling tired and exhausted, thinking about difficulty concentrating, thinking about feeling passive and unmotivated, thinking "why can't I get going?", and thinking about not getting things done better. Participants responded on a scale from 1 (never) to 4 (always) with higher scores indicating more rumination. Internal consistency in the present study was good, Cronbach's $\alpha = 0.85$. To probe rumination in the context of positive mood, participants filled in the Responses to Positive Affect (RPA) Questionnaire (Feldman et al., 2008). The 17-item questionnaire is divided into three subscales: dampening (i.e., mental strategies to attenuate positive mood; 8 items), self-focused positive rumination (4 items), and emotion-focused positive rumination (5 items). Each item is answered on a scale from 1 (almost never) to 4 (almost always), i.e., higher scores indicate greater endorsement of each subscale. In the present study, internal consistency was acceptable for the subscale dampening (Cronbach's $\alpha = 0.78$), good for self-focused positive rumination (Cronbach's $\alpha = 0.82$), and acceptable for emotion-focused positive rumination (Cronbach's $\alpha = 0.63$).

With regard to emotional processing, we assessed participants' ability to regulate emotions using the Emotion Regulation Questionnaire (Gross & John, 2003). Six items index habitual use of cognitive reappraisal and the remaining four items measure expressive suppression. All items are answered on a scale from 1 (strongly disagree) to 7 (strongly agree) and we calculated the respective sum scores. In the present study, internal consistency was good for the subscale reappraisal (Cronbach's $\alpha = 0.87$) and acceptable for suppression (Cronbach's $\alpha = 0.70$).

Furthermore, we measured dispositional mindfulness with the Mindfulness Attention and Awareness Scale (MAAS; Brown & Ryan, 2003; Michalak et al., 2008). 15 items describe experiences of mindlessness (e.g., "I find myself doing things without paying attention"), each answered on a scale ranging from 1 (almost always) to 6 (almost never). Higher scores therefore indicate greater mindfulness. Internal consistency in the present study was excellent (Cronbach's $\alpha = 0.92$). Optimism was indexed using the total score of the revised Life Orientation Test (LOT-R; Glaesmer et al., 2018; Scheier & Carver, 1985) which sums the optimism scale (3 items) and the inverted pessimism scale (3 items). Items describe e.g., positive beliefs regarding one's future and are answered on a scale from 0 (strongly disagree) to 4 (strongly agree) with higher scores indicating greater optimism. Internal consistency in the present study was acceptable, Cronbach's $\alpha = 0.70$.

Supplement 2. Details on saliva sampling and genotyping

DNA for genotyping was isolated from saliva samples collected in OG-500 Oragene kits (DNA Genotek, Ottawa, Ontario, Canada) according to the manufacturer's instructions. Genotyping was performed by technical personnel of the Division of Molecular Neuroscience, University of Basel, Switzerland, blinded to any other data.

Genotyping of the *BCL1* polymorphism was done with Pyrosequencing on a PyroMark™ID System (Biotage, Uppsala, Sweden). Primers were: 5'-CTT GCA GAA GAG GAT TCA CAT CA-3' (forward, 5'- biotinylated), 5'- GTG TGT CTG CCT GAA GGA ATG-3' (reverse), 5'- GTG TAT CTC AGA AAA GAC CT-3' (sequencing primer). Genotyping of the *KIBRA* rs17070145 polymorphism was also done with Pyrosequencing. Primers were: 5'- ACA CCT CTG TGG CTT TTC TCC -3' (forward), 5'- ACA AGG CTG TGG AAT CTC TTG A -3' (reverse, 5' biotinylated), 5'- CCT TGA TCC TGG ACC -3' (sequencing primer). Genotyping of the *ADRA2B* deletion polymorphism was done by PCR and visualization of the bands on an agarose gel. Forward primer: 5'-AGA AGG AGG GTG TTT GTG GGG-3', reverse primer: 5'-ACC TAT AGC ACC CAC GCC CCT-3', annealing temperature 58°C.

Supplement 3. Sensitivity analyses

Sensitivity analyses were conducted to determine the smallest effect sizes detectable with 80% power for the regression models predicting mental health outcomes and work problems from intrusion occurrence (Supplementary Tables 5-9). Analyses were performed using the *pwr.f2.test()* function from the *pwr* R package (Champely, 2020). Given the sample sizes across models (N = 87–124) and α = .05, the minimal detectable partial R^2 values ranged from .062 to .086. For the regression model predicting work problems at T2, the minimal detectable partial R^2 was .062, and the observed partial R^2 for intrusion occurrence was .08, indicating adequate power to detect this primary effect of interest. For models predicting depression and anxiety, the observed partial R^2 values for intrusion occurrence were below the minimum detectable effect threshold, suggesting limited power to reliably detect such small effects. The R code and HTML output for all sensitivity analyses are available on the OSF.

Champely, S. (2020). *pwr: Basic functions for power analysis* (R package version 1.3-0) [Computer software]. https://doi.org/10.32614/CRAN.package.pwr

Supplementary Tables

Supplementary Table 1. Overview of included predictors

Construct	Measure
Demographics	Sex (m/f)<br>Age in years
Prior trauma	Posttraumatic Diagnostic Scale<br>(PDS; Foa et al., 1997)
Cognitive & emotional processing	
Cognitive flexibility	Cognitive Flexibility Questionnaire<br>(Martin & Rubin, 1995)
Rumination	Response Styles Questionnaire<br>(Nolen-Hoeksema, 1991)
Responses to positive affect	Responses to Positive Affect Questionnaire, Subscales:<br>- dampening<br>- self-focused positive rumination<br>- emotion-focused positive rumination<br>(Feldman et al., 2008)
Emotion regulation	Emotion Regulation Questionnaire, Subscales:<br>- reappraisal<br>- suppression<br>(Gross & John, 2003)
Mindfulness	Mindfulness Attention and Awareness Scale<br>(Brown & Ryan, 2003; Michalak et al., 2008)
Optimism	Life Orientation Test<br>(Glaesmer et al, 2018; Scheier & Carver, 1985)
Memory-related gene polymorphisms	BCL1 polymorphism of *NRC31* gene<br>ADRA2B gene deletion variant<br>KIBRA gene polymorphism

Supplementary Table 2. Zero-order correlations between predictors of intrusion occurrence

	(1)	(2)	(3)	(4)	(5)	(6)	(7)	(8)	(9)	(10)	(11)
(1) Mindfulness	1										
(2) Rumination	-0.16	1									
(3) Prior trauma	0.11	-0.03	1								
(4) Cognitive flexibility	**0.24***	**-0.33*****	0.05	1							
(5) Reappraisal	-0.10	**-0.24***	-0.03	**0.28****	1						
(6) Suppression	-0.04	**0.21***	0.00	**-0.27****	-0.03	1					
(7) Emotion-focused positive rumination	**-0.22***	-0.03	0.05	**0.29****	**0.24***	**-0.25***	1				
(8) Dampening	-0.05	**0.42*****	-0.15	**-0.32****	**-0.21***	**0.21***	-0.07	1			
(9) Self-focused positive rumination	-0.13	-0.16	-0.16	**0.34*****	**0.26****	-0.13	**0.61*****	-0.07	1		
(10) Optimism	-0.15	**0.51*****	0.08	**-0.43*****	**-0.27****	**0.38*****	**-0.26****	**0.33****	**-0.38*****	1	
(11) Age	**0.54*****	-0.13	0.15	0.07	0.02	0.05	**-0.23***	0.05	**-0.20***	-0.09	**1**

Note. * $p < 0.05$, ** $p < 0.01$, *** $p < 0.001$

Supplementary Table 3. Predicting intrusion-related distress at 3-months follow-up

	β	*SE*	*std. Beta*	*95% CI*	*T*	*p*
(Intercept)	2.98	1.04	0.00	0.90 – 5.06	2.87	**0.006**
Cognitive Flexibility	-0.02	0.01	-0.19	-0.05 – 0.01	-1.42	0.161
Rumination	0.04	0.03	0.21	-0.01 – 0.09	1.63	0.109
Emotional Suppression	-0.12	0.11	-0.15	-0.34 – 0.09	-1.16	0.249
Observations	57					
R^2 / R^2 adjusted	0.111 / 0.061					

Supplementary Table 4. Mental health and work problems by time point

	**T0**	**T1**	**T2**
Anxiety	6 (3.06)	13 (1.76)	13 (1.80)
Depression	3 (2.56)	3 (2.80)	3 (2.74)
Work Problems	-	2 (0.46)	2 (0.45)

Note. Values show the mean with standard deviation in parentheses.

Supplementary Table 5. Association between depressive symptoms at T1 and intrusion occurrence at T1

	β	*SE*	*std. Beta*	*95% CI*	*T*	*p*
(Intercept)	0.15	1.61	-0.20	-3.05 – 3.36	0.09	0.925
Intrusion T1 (yes)	0.62	0.54	0.21	-0.46 – 1.70	1.14	0.258
Depression T0	0.74	0.34	0.54	0.06 – 1.42	2.16	**0.034**
Age	0.01	0.05	0.01	-0.09 – 0.11	0.11	0.910
Sex (male)	-0.37	0.76	-0.13	-1.89 – 1.15	-0.48	0.630
Observations	88					
R^2 / R^2 adjusted	0.521 / 0.498					

Note. Sensitivity power analysis indicated that, given the number of observations for this model, only moderate-to-large effects (partial $R^2 \geq .086$) could be reliably detected with 80% power ($\alpha = .05$). The observed partial R^2 for intrusion occurrence was .015, well below this threshold. See our OSF repository for full R code and sensitivity analysis outputs.

Supplementary Table 6. Association between depressive symptoms at T2 and intrusion occurrence at T2

	β	*SE*	*std. Beta*	*95% CI*	*T*	*p*
(Intercept)	0.30	1.06	-0.19	-1.80 – 2.40	0.28	0.779
Intrusion T2 (yes)	0.50	0.47	0.19	-0.43 – 1.44	1.07	0.286
Depression T0	0.54	0.13	0.44	0.29 – 0.79	4.29	**<0.001**
Age	0.01	0.04	0.02	-0.06 – 0.08	0.33	0.739
Sex (male)	-0.24	0.41	-0.09	-1.05 – 0.57	-0.58	0.561
Observations	104					
R^2 / R^2 adjusted	0.321 / 0.293					

Note. Sensitivity power analysis indicated that, given the number of observations for this model, only moderate-to-large effects (partial $R^2 \geq .073$) could be reliably detected with 80% power ($\alpha = .05$). The observed partial R^2 for intrusion occurrence was .011, well below this threshold. See our OSF repository for full R code and sensitivity analysis outputs.

Supplementary Table 7. Association between anxiety symptoms at T1 and intrusion occurrence at T1

	β	*SE*	*std. Beta*	*95% CI*	*T*	*p*
(Intercept)	13.10	1.17	0.07	10.78 – 15.42	11.23	**<0.001**
Intrusion T1 (yes)	0.08	0.38	0.05	-0.68 – 0.84	0.21	0.833
Anxiety T0	-0.21	0.06	-0.38	-0.33 – -0.10	-3.74	**<0.001**
Age	0.03	0.04	0.10	-0.04 – 0.10	0.96	0.339
Sex (male)	-0.29	0.37	-0.17	-1.02 – 0.44	-0.79	0.429
Observations	87					
R^2 / R^2 adjusted	0.143 / 0.101					

Note. Sensitivity power analysis indicated that, given the number of observations for this model, only moderate-to-large effects (partial $R^2 \geq .087$) could be reliably detected with 80% power ($\alpha = .05$). The observed partial R^2 for intrusion occurrence was .001, well below this threshold. See our OSF repository for full R code and sensitivity analysis outputs.

Supplementary Table 8. Association between anxiety symptoms at T2 and intrusion occurrence at T2

	β	*SE*	*std. Beta*	*95% CI*	*T*	*p*
(Intercept)	13.99	0.73	0.27	12.53 – 15.44	19.07	**<0.001**
Intrusion T2 (yes)	-0.32	0.31	-0.18	-0.94 – 0.31	-1.00	0.318
Anxiety T0	-0.21	0.05	-0.35	-0.31 – -0.11	-4.16	**<0.001**
Age	0.02	0.02	0.05	-0.02 – 0.06	0.94	0.349
Sex (male)	-0.83	0.36	-0.47	-1.55 – -0.11	-2.31	**0.023**
Observations	95					
R^2 / R^2 adjusted	0.201 / 0.166					

Note. Sensitivity power analysis indicated that, given the number of observations for this model, only moderate-to-large effects (partial $R^2 \geq .08$) could be reliably detected with 80% power ($\alpha = .05$). The observed partial R^2 for intrusion occurrence was .011, well below this threshold. See our OSF repository for full R code and sensitivity analysis outputs.

Supplementary Table 9. Association between work problems at T2 and intrusion occurrence at T2

	β	*SE*	*std. Beta*	*95% CI*	*T*	*p*
(Intercept)	1.07	0.23	-0.24	0.61 – 1.53	4.58	**<0.001**
Intrusion T2 (yes)	0.25	0.08	0.58	0.10 – 0.41	3.21	**0.002**
Work Problems T1	0.46	0.11	0.49	0.25 – 0.66	4.32	**<0.001**
Age	-0.00	0.01	-0.03	-0.02 – 0.01	-0.35	0.727
Sex (male)	0.05	0.08	0.12	-0.10 – 0.20	0.71	0.479
Observations	124					
R^2 / R^2 adjusted	0.336 / 0.314					

Note. Sensitivity power analysis indicated that, given the number of observations and $\alpha = .05$, this model had 80% power to detect a partial $R^2 \geq .062$. The observed partial R^2 for intrusion occurrence was .08, indicating adequate power to detect the primary effect of interest. See our OSF repository for full R code and sensitivity analysis outputs.

Supplementary Table 10. Association between work problems at T2 and intrusion occurrence at T2 with baseline mental health as covariates

	β	*SE*	*std. Beta*	*95% CI*	*T*	*p*
(Intercept)	0.60	0.34	-0.26	-0.07 – 1.28	1.79	0.078
Intrusion T2 (yes)	0.23	0.09	0.59	0.05 – 0.41	2.56	**0.013**
Work Problems T1	0.49	0.10	0.60	0.30 – 0.68	5.09	**<0.001**
Depression T0	-0.02	0.03	-0.12	-0.09 – 0.04	-0.74	0.463
Anxiety T0	0.04	0.02	0.28	-0.00 – 0.08	1.92	0.060
Age	0.00	0.01	0.05	-0.01 – 0.02	0.45	0.653
Sex (male)	0.15	0.09	0.39	-0.03 – 0.34	1.64	0.106
Observations	70					
R^2 / R^2 adjusted	0.588 / 0.548					
